# Exploratory single‐nucleus multiomics analysis of myeloid cell states associated with neoadjuvant chemotherapy response in pancreatic ductal adenocarcinoma

**DOI:** 10.1002/ctm2.70810

**Published:** 2026-09-14

**Authors:** Mercy Ojetunde, Li Ma, Hillary G. Goodney, Alyson M. Stevens, Alan Mizener, Emidio E. Pistilli, Sascha Ott, Kawther Abdilleh, Barbara Szomolay, Gangqing Hu, Timothy D. Eubank, Brian A. Boone

**Affiliations:** ^1^ Department of Microbiology Immunology and Cell Biology West Virginia University School of Medicine Morgantown West Virginia USA; ^2^ Cancer Cell Biology West Virginia University School of Medicine Morgantown West Virginia USA; ^3^ Department of Human Performance ‐ Exercise Physiology West Virginia University School of Medicine Morgantown West Virginia USA; ^4^ Warwick Medical School University of Warwick Coventry UK; ^5^ Pancreatic Cancer Action Network El Segundo California USA; ^6^ Division of Infection and Immunity & Systems Immunity Research Institute Cardiff University Cardiff UK; ^7^ WVU Cancer Institute West Virginia University Morgantown West Virginia USA; ^8^ Department of Dermatology West Virginia University School of Medicine Morgantown West Virginia USA; ^9^ Department of Surgery West Virginia University School of Medicine Morgantown West Virginia USA

**Keywords:** lipid‐associated macrophages (LAMs), myeloid cell heterogeneity, neoadjuvant chemotherapy, pancreatic ductal adenocarcinoma (PDAC), single‐cell multiomics, tumour microenvironment, tumour‐associated macrophages (TAMs)

## Abstract

**Background:**

Pancreatic ductal adenocarcinoma (PDAC) continues to be one of the most lethal human malignancies, with the vast majority of patients ineligible for immunotherapy. Tumour‐associated macrophages (TAMs) are key regulators of the PDAC tumour microenvironment (TME), yet their transcriptional and epigenomic heterogeneity in the context of chemotherapy response is poorly understood. Therefore, we performed an exploratory single nucleus multiomics analysis of PDAC tumors stratified by histopathologic response to neoadjuvant chemotherapy.

**Methods:**

Surgical resection specimens from PDAC patients were classified as responders or non‐responders using the American College of Pathologists (CAP) histopathologic criteria. Frozen tissue underwent simultaneous snRNA‐seq and snATAC‐seq on the 10x Genomics Chromium Single Cell Multiome platform, followed by downstream analyses such as differential gene expression, GO and hallmark pathway enrichment, pseudotime trajectory inference and ChromVAR transcription factor motif analysis.

**Results:**

Multiomics profiling of 30 840 high‐quality nuclei revealed a myeloid compartment that differed in composition and transcriptional state between CAP‐defined responders and non‐responders in this small cohort. We observed a trend toward higher LAM‐like state proportions in the responders than non‐responders (38.4% vs. 26.7%), although this disparity did not achieve statistical significance. The transcriptional programs of the responder myeloid cells are associated with phagocytosis and lipid handling. Chromatin accessibility analysis further suggested candidate response‐associated transcription factor motif accessibility patterns.

**Conclusions:**

Neoadjuvant‐treated PDAC tumours from CAP‐defined responders in this cohort myeloid landscape with apparent enrichment of LAM‐like states and immune‐activating transcriptional/epigenetic programs. However, these findings are preliminary and hypothesis‐generating because of the small cohort size, heterogeneous treatment regimens, absence of matched pre‐treatment biopsies, and lack of knockout validation. Larger treatment cohorts and functional/mechanistic studies are needed to determine whether LAM‐like myeloid programs contribute to chemotherapy response or reflect a consequence of chemotherapy treatment.

## Graphical abstract

- Neoadjuvant‐treated PDAC tumors were stratified into responders and non‐responders based on CAP guidelines
- snRNA‐seq, snATAC‐seq, and spatial transcriptomics can be used to profile the neoadjuvant‐chemotherapy‐treated PDAC immune landscape.
- Our exploratory findings nominate candidate myeloid features for validation in larger cohorts and functional studies.

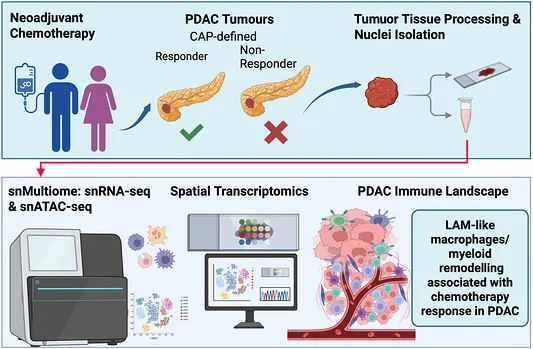

## 1 INTRODUCTION

Pancreatic ductal adenocarcinoma (PDAC) is among the deadliest human cancers,^1^ with a 5‐year survival rate of only 13%. PDAC has emerged as the third leading cause of cancer‐related deaths in the United States, with a continuous rise in incidence, and is anticipated to become the second leading cause of death by 2030.^2^, ^3^ Despite significant advancements in therapeutic strategies in recent decades, developing effective treatments for PDAC remains a major challenge because of its complex tumour microenvironment (TME).^4^ This TME is characterized by tumour‐infiltrating immune cells,^5^ immunosuppressive cells,^6^ and cancer‐associated fibroblasts (CAFs),^7^ which promote immunosuppression,^8^ disease progression, dense desmoplasia,^9^ resistance to therapy, and metastatic dissemination.^10^, ^11^ The characteristics of the PDAC TME not only hinder the efficacy of conventional and emerging therapies, but also highlight the urgent need to apply advanced technologies to fully characterize and better understand this complex milieu to enable novel interventions to reprogram the aggressive microenvironment.

Immune components, such as myeloid cells—including tumour‐associated macrophages (TAMs), tumour‐associated neutrophils (TANs), dendritic cells and myeloid‐derived suppressor cells (MDSCs), comprise a functionally diverse compartment of the TME capable of exerting both pro‐tumorigenic and anti‐tumour effects depending on their phenotypic state and microenvironmental cues.^12^, ^13^ Among these, macrophages rank among the most prevalent immune cells in the TME and are known for their plasticity/heterogeneity to polarize into distinct subsets.^14^, ^15^ Classically activated M1 macrophages, characterized by markers such as IL‐6, TNF‐α and IL‐1β, are proinflammatory and immunostimulatory, generating ROS‐induced oxidative damage and anti‐tumoral activity. Conversely, alternatively activated M2 macrophages produce anti‐inflammatory cytokines such as IL‐10 and TGF‐β and are crucial in inflammation resolution, tissue remodelling, and tumour progression.^16^, ^17^ TAMs in solid tumours, including PDAC, are often skewed towards an M2‐like phenotype,^18^ and are broadly considered to be immunosuppressive and tumour‐promoting. However, emerging evidence suggests that TAMs are not homogeneous; instead, they exist as plastic and diverse subsets with continuous, dynamic polarization, distinct metabolic, transcriptional, and functional profiles,^19^ some of which may support anti‐tumour immune responses.^20^, ^21^ Given these complexities and the current failure of meaningful treatment effects of immunotherapy regimens in PDAC,^8^, ^22^, ^23^ there is an urgent need to comprehend how the immunologically ‘cold’ PDAC TME influences chemotherapy response, with the goal of uncovering mechanisms that could enhance treatment success.^10^, ^24^ Accordingly, the central question motivating this study is: which tumour‐infiltrating myeloid cell states, and their associated transcriptional and regulatory programs, distinguish PDAC patients who respond to neoadjuvant chemotherapy from those who do not, and whether these programs are associated with immune microenvironments linked to chemotherapy response or resistance.

Because myeloid cells integrate stromal, metabolic, and damage‐associated cues in PDAC, we further reasoned that chemotherapy response may be associated with specific macrophage programs linked to tissue remodelling, immunoregulation, lipid handling and scavenger functions, features that have recently emerged as hallmarks of distinct TAM states across tumour types.^25^ Recent advances in PDAC studies have involved the use of single‐cell RNA‐sequencing (scRNA‐seq), ATAC‐seq, and integrative multiomics profiling to identify distinct immune cell subsets in PDAC,^26^, ^27^ prognostic markers,^28^, ^29^, ^30^ and treatment‐associated changes in the PDAC tumour microenvironment.^29^, ^31^ While these studies have been informative, none have analysed cohorts with disparate chemotherapy responses in human PDAC, which are frequently seen in clinical practice without clear contributing factors or mechanisms for these contrasting responses. This represents a significant clinical challenge and gap in the field, as clinicians cannot predict patients’ responses to new combination therapies to guide treatment selection, underscoring the need to identify determinants of therapeutic response and resistance. In this current exploratory study, we profiled PDAC tumours from neoadjuvant‐treated patients classified as responders or non‐responders by histopathologic treatment‐response criteria. Our results suggest response‐associated myeloid heterogeneity, including a higher average proportion of lipid‐associated macrophages (LAMs) in responders. LAMs are a subset of tumour‐associated macrophages defined by lipid accumulation, characterized by the expression of genes such as *GPNMB*, *CD9* and *APOE*.^19^ While LAMs have been implicated in immunosuppression and tumour progression in some cancers like breast and prostate cancers,^32^, ^33^, ^34^ some studies have reported the immunoprotective role of LAM‐like macrophages, such as TREM2+ LAMs.^35^, ^36^, ^37^ Therefore, we interpret our observation with caution and propose that LAM‐like macrophage states in chemotherapy‐treated PDAC may represent a context‐dependent, therapy‐associated phenotype that requires further validation.

## 2 MATERIALS AND METHODS

### 2.1 Patient tissue and data collection

Prior to sample collection, Institutional Review Board approval was obtained from West Virginia University (#1903496995), and all patients signed informed consent. All patients underwent surgical resection with pancreaticoduodenectomy as part of the standard of care. PDAC tissue samples from responders and non‐responders were obtained from the WVU Biospecimen and Translational Research Analysis Core (BioTRAC) upon surgical resection. Treatment response was determined using the American College of Pathologists (CAP) guidelines,^38^, ^39^ which score treatment effect according to the extent of residual tumour present after therapy. Patients with CAP grade 2 were classified as partial responders, whereas patients with CAP grade 3 were classified as non‐responders; no CAP 0 case was included in this cohort, and although we had one CAP 1 case, it was too small to draw conclusions, so we excluded it from the study cohort. Because CAP grade may not fully capture long‐term clinical benefit in PDAC, response‐group comparisons in this study are interpreted as histopathology‐defined and hypothesis‐generating rather than definitive clinical‐response biomarkers. PDAC tumour tissue was either frozen independently for nucleus isolation or embedded in OCT (optimal cutting temperature compound) and subsequently stored at −80°C. Clinicopathological variables including sex, age, BMI, chemotherapy type, tumour stage (TNM classification), overall survival, pre‐NAT CA19‐9 and pre‐operative CA19‐9 were collected for each patient and reported to the investigative team in a blinded fashion.

### 2.2 Nuclei isolation from frozen tissue

Nuclei were isolated from frozen tissue employing a protocol adapted from the Chromium Nuclei Isolation from Complex Tissues for Single Cell Multiome ATAC + Gene Expression Sequencing (10x Genomics, CG000375). Approximately 50 mg of frozen tissue was sectioned into five fragments on ice and homogenized for 1.5 min using a tissue homogenizer in ice‐cold lysis buffer (10 mM Tris–HCl, 10 mM NaCl, 3 mM MgCl_2_, 0.1% Nonidet P40 substitute, 1 mM dithiothreitol (DTT) in nuclease‐free water). Homogenates were incubated on ice for 5 min and filtered through a 70 µm cell strainer. Suspensions underwent centrifugation at 500 × g for 5 min at 4°C. Pellets were resuspended in 1 mL of 1% bovine serum albumin (BSA) in DPBS and incubated on ice for 5 min prior to gentle resuspension and a second centrifugation (500 × g, 5 min, 4°C). Nuclei were resuspended in 1 mL 1% BSA in DPBS and filtered through a 30 µm cell strainer to obtain a single‐nucleus suspension. All steps were performed on ice. Nuclei were stained with 7‐aminoactinomycin D (7‐AAD) 1:100 readymade solution (Sigma, SML‐1633) and sorted using a FACSAria III cell sorter (BD Biosciences). Sorted nuclei were collected into 10% BSA. For snMultiome, RNase inhibitor of 1 U/mL (Sigma–Aldrich, 03335402001) was added to all final solutions.

### 2.3 snMultiome library preparation

Single‐nucleus Multiome libraries were generated following the manufacturer's protocol using the Chromium Single Cell Multiome ATAC + Gene Expression kit (10x Genomics, 1000283). Sorted nuclei were pelleted (500 × g, 5 min, 4°C) and resuspended in 100 µL of 0.1× lysis buffer (10 mM Tris–HCl, 10 mM NaCl, 3 mM MgCl_2_, 1% BSA, 1 U/mL RNase inhibitor, 1 mM DTT, 0.01% Tween‐20, 0.01% Nonidet P40 substitute, and 0.001% digitonin in nuclease‐free water) and incubated on ice for 2 min. Nuclei were washed with 1 mL wash buffer (10 mM Tris–HCl, 10 mM NaCl, 3 mM MgCl_2_, 1% BSA, 1 U mL^−^^1^ RNase inhibitor, 0.1% Tween‐20, and 1 mM DTT in nuclease‐free water) followed by centrifugation (500 × g, 5 min, 4°C). The pellet was resuspended in 1× nuclei buffer supplemented with 1 U/mL RNase inhibitor and 1 mM DTT. ATAC and cDNA reactions were performed according to the manufacturer's instructions. Nuclei were loaded onto a Chromium Next GEM Chip J (10x Genomics, 1000230) and processed on the Chromium Controller, targeting 10 000 nuclei per reaction. We assessed library quality using an Agilent Bioanalyzer. Libraries were sequenced on an Illumina NextSeq 2000 sequencer at the Marshall University Genomics Core Facility.

### 2.4 Quality control and filtering

snMultiome short sequencing reads were aligned to the human reference genome (hg38) and quantified using Cell Ranger‐arc count (version 2.0.2). For each sample, the minimum feature threshold was set as nCount_ATAC (number of ATAC‐seq fragments) > 400 and nCount_RNA (number of RNA fragments) > 400 and nFeature_ATAC (number of ATAC‐seq peaks) > 200 and nFeature_RNA (number of genes) > 200. The maximum feature threshold was determined based on its individual feature distribution. The detailed thresholds for all samples are provided in Supporting Information. For other features, all samples use the common threshold as blacklist_fraction < 2%, nucleosome_signal < 1.5, and 2 < TSS.enrichment < 10. Although a 5% mitochondrial RNA cutoff is commonly used for scRNA‐seq, we selected a stricter percent.mt threshold empirically because this snMultiome PDAC dataset exhibited uniformly low mitochondrial RNA content (approximately the 95th percentile was < 1% across samples). Therefore, percent.mt < 1% was used to remove only the extreme high‐mitochondrial tail events most consistent with compromised nuclear integrity/ambient RNA, while retaining the vast majority of high‐quality nuclei. Other parameters included percent.mt.atac (ATAC‐seq reads mapping to mitochondrial DNA) < 5%, percent.ribo (reads from ribosomal genes) < 2%, and pct_reads_in_peaks (fraction of fragments within ATAC‐Seq peaks) > 10%. Nucleosome_signal (strength of the nucleosome signal), TSS.enrichment (transcription start site [TSS] enrichment score as defined by ENCODE) and blacklist_fraction (ratio reads in genomic blacklist regions as defined by ENCODE) were calculated by the function NucleosomeSignal, TSSEnrichment and FractionCountsInRegion of Signac, respectively. Percent.mt and percent.ribo were calculated by the PercentageFeatureSet function. percent.mt.atac was calculated based on 100*atac_mitochondrial_reads/atac_raw_reads.

### 2.5 snMultiome data pre‐processing and clustering analysis

After quality control of the snMultiome data, Seurat (version 5.0.1)^40^ and Signac (version 1.12.0)^41^ were used for downstream analysis. For the RNA component of the snMultiome data, NormalizeData was used for normalization, and the normalization method was set to ‘CLR’ (centred log ratio transformation). The cell cycle phase score was calculated by CellCycleScoring. Variable features were identified by FindVariableFeatures and the top 2000 were selected. Data were scaled and centred by the ScaleData function and regressed out the effects of cell cycle, nCount_RNA, nCount_ATAC, percent.mt, percent.mt.atac and percent.ribo. RunPCA was used for principal component analysis (PCA) dimensionality reduction. We used RunHarmony to integrate the dataset. RunUMAP was used for dimensional reduction and visualization via uniform manifold approximation and projection (UMAP). We used FindNeighbours to compute nearest neighbours for the object. FindClusters was used to identify clusters of cells by a shared nearest neighbours modularity optimization based on original Louvain algorithm with a resolution of 0.2. For the ATAC component of the snMultiome data, RunTFIDF was used to compute term‐frequency inverse‐document‐frequency normalization on the matrix. FindTopFeatures was used to find the most frequently observed features. RunSVD was used to find the top 50 largest singular values and corresponding singular vectors of the matrix. RunHarmony was used to correct batch effects. RunUMAP was used for dimensional reduction and visualization via uniform manifold approximation and projection (UMAP). FindClusters was used to identify cell clusters using shared nearest neighbours modularity optimization based on Smart Local Moving (SLM) clustering algorithms, with a resolution of 0.2.

### 2.6 snMultiome subcluster's markers and differentially expressed genes identification

Marker genes for each subcluster were identified using the FindAllMarkers function with the Wilcoxon rank‐sum test. Parameters included min.pct = 0.1, only.pos = TRUE, and logfc.threshold = 0.25, using the RNA sequencing part from the scMultiome data. After cell types were assigned based on subcluster specific marker genes, cell type marker genes were also identified using the FindAllMarkers function with the same parameters.

Differentially expressed genes associated with different response types within each cell type were identified using the FindMarkers function with the Wilcoxon rank‐sum test, using min.pct = 0.1, logfc.threshold = 0.25, return.thresh = 0.01 and the RNA assay. These analyses were used primarily for exploratory marker discovery and pathway nomination. Cell‐type and myeloid‐subset proportions were compared at the patient‐sample level where possible, whereas cell‐level differential expression results were interpreted with caution because individual nuclei from the same patient are not fully independent biological replicates. Due to the limited sample size, formal pseudobulk modelling with robust adjustment for treatment regimen, baseline clinical features and donor‐level random effects was underpowered; therefore, response‐associated transcriptional patterns are presented as hypothesis‐generating and require validation in larger cohorts.

### 2.7 Myeloid sub‐population identification

Myeloid population was extracted using the subset function based on cell type annotation. For the extracted object, we did the similar procedures describe as in 2.5 and 2.6 part to identify its subclusters and marker genes. FindClusters's resolution was set as 0.5.

### 2.8 ChromVAR analysis of snATAC‐Seq dataset

For the ATAC component of the snMultiome data, transcription factor motif enrichment scores were calculated using the RunChromVAR^42^ function in Signac (version 1.12.0).^41^ Motif position weight matrices were obtained from the JASPAR2020 database. For each cell type and its corresponding response type, the average enrichment score was computed by aggregating cell‐wise scores and dividing by the number of cells in the group. To identify cell type‐specific and response type‐specific motifs, the FindAllMarkers function from the Signac package was applied with the following parameters: only.pos = TRUE, mean.fxn = rowMeans, and fc.name = 'avg_diff'. Finally, motifs with an adjusted *p*‐value < .01 were identified as specific motifs.

### 2.9 GO enrichment analysis

GO enrichment analysis was performed using Metascape,^43^ with expressed genes detected in at least 1% of cells used as the background set (Supporting Information). We selected the top 10 hits for visualization.

### 2.10 Trajectory analysis

After myeloid sub‐populations were identified, we used the monocle3^44^ package to build the development trajectory of the myeloid subpopulations. Learn_graph was used to learn the trajectory. Pseudotime feature was added to the Seurat object for UMAP visualization. The root assignment was based on prior biological knowledge, specifying dendritic cells as the starting population.

### 2.11 Spatial transcriptomics

PDAC tumour specimens, which include a chemotherapy responder PDAC sample and an untreated PDAC sample, were fixed and embedded in paraffin blocks and maintained at −80°C until they were sectioned. The blocks were equilibrated to the cryostat chamber (−20°C). Tissue was sectioned at 10 µm and mounted onto positively charged glass slides compatible with the 10x Genomics Visium CytAssist workflow. For the FFPE samples, 4 × 10 ‐µm FFPE sections were obtained for RNA extraction using the RNeasy FFPE Kit (Qiagen, 73504). DV200 of the extracted RNA was evaluated using Agilent RNA 6000 Pico Kit. Samples with DV200 > 30% were selected for sectioning, deparaffinization, and H&E staining on the Visium Spatial Slide with capture area of 6.5 × 6.5 mm^2^. Probe hybridization, probe ligation, probe release and extension, and library construction were performed in accordance with the Visium Spatial Gene Expression Reagent Kit for FFPE User Guide (10x Genomics, CG000407 Rev A). To capture the area of interest, the samples were selected for Visium CytAssist, which facilitates the transfer of transcriptomic probes from standard glass slides to Visium slides, enabling whole‐transcriptomic spatial profiling. Briefly, the slides were subjected to H&E staining and imaged at ×20 magnification. The Visium CytAssist instrument and a disposable fiducial frame were used to align stained sections on standard glass slides, which were then transferred to the capture area of a Visium Gene Expression slide according to our WVU Flow Cytometry & Single Cell Core Facility's SOP adapted directly from the 10x Genomics manual (CG000495, RevB). Representative regions with at least 2 nerves within a 6.5 × 6.5 mm^2^ area were selected by an appropriate configuration that allows the tissue slide gasket to encompass the region of interest. Then the coverslips were removed, and the slides were destained by 0.1N HCl and decrosslinked by Diluted Decrosslinking Buffer at 95°C for 1 h (10x Genomics, CG000658, RevA). Probe hybridization and probe ligation were conducted according to the user guide (10x Genomics, CG000495, RevB). Then the hybridized probes were released and transferred to a Visium CytAssist Spatial Gene Expression Slide. After transfer, probes were extended, eluted and pre‐amplified. Then, the Sample Index PCR was applied to construct the cDNA library. The libraries were sequenced on an Illumina NextSeq 2000 sequencer at the Marshall University Genomics Core Facility.

### 2.12 Spatial transcriptomics data processing

Primary analysis was performed on each sample individually using the 10x Genomics Space Ranger (Version 3.1.2; RRID:SCR_025848) count function. The prebuilt 10x genomics GRCh38 reference was used for the transcriptome reference, the 10x Visium Human Transcriptome Probe Set v2.0 was used for the probe set, and the 10x Visium Human Immune Profiling Panel v1.0 was used for the feature reference.

Secondary analysis was performed in Python using Scanpy^45^ (Version 1.11.4; RRID: SCR_018139) and Squidpy^45^ (Version 1.6.2; RRID: SCR_026157) with code available at (https://github.com/PistilliLab/Boone_10x_Visium_Analysis). In brief, both libraries were aggregated and pre‐processed using the quality control functions in Scanpy. Spatial data were filtered to remove spots where gene expression data (GEX) had less than 100 counts, less than 100 unique genes, or greater than 10% of counts mapping to mitochondrial genes. Antibody capture data were then filtered to spots shared with the filtered GEX data. Preprocessing including, count normalization, log transformation and scaling were performed sequentially using the appropriate functions in Scanpy. Technical variance was removed using Scanpy's regress_out method over the total counts, number of unique genes, and percentage of mitochondrial counts. The top 500 most variable genes were selected using the Seurat flavor of Scanpy's highly_variable_genes method. Scanpy's implementation of Harmony^46^ (RRID: SCR_022798) was used for batch effect correction prior to clustering. Scaled expression of the top 500 most variable genes was used for clustering with the Leiden algorithm^47^ at a resolution of .6. Cluster annotations were then applied manually based on projected cell‐type annotations from Tangram^48^ using single‐cell reference data from Peng J et al.^49^ Cluster annotations were then validated by manually inspecting the top 10 marker genes for each cluster.

Within the selected myeloid subset, we examined the expression of LAM genes (*APOE, APOC1, PPARG, GPNMB, GLUL, SNTB1, MITF*) identified in our multiomics data to visually compare LAM gene expression between a PDAC chemotherapy responder and untreated PDAC tissue. Spatial gene expression patterns were visualized by UMAP projections, spot overlay plots, and violin plots.

### 2.13 Statistical Considerations and Interpretation Framework

Considering the limited sample size and the nested structure of single‐nucleus data, all response‐associated analyses were interpreted within an exploratory framework. Patient‐level proportions were used when comparing cell‐type or myeloid‐subset abundance between responders and non‐responders.

## 3 RESULTS

### 3.1 Single‐cell transcriptomic landscape of pancreatic tumours suggest cellular diversity and response‐associated differences in cell composition

**FIGURE 1:**
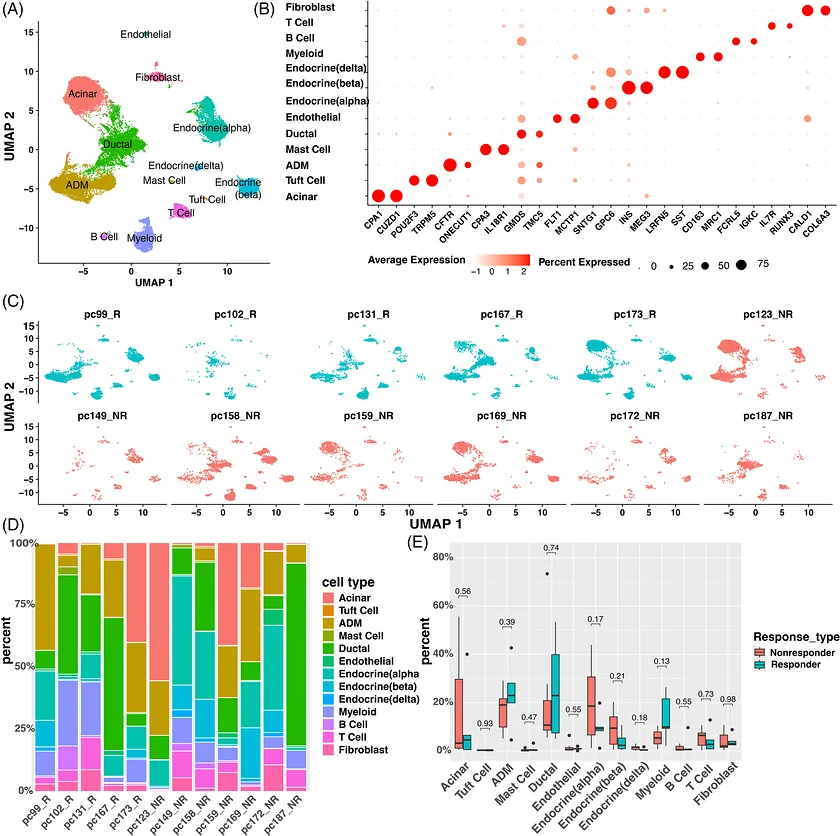
Single‐nucleus transcriptomic landscape and cell‐type composition of neo‐adjuvant‐treated PDAC tumours. (A) UMAP visualization of cell type annotation based on the single cell gene expression from the scMultiome data of PDAC tumours. (B) Dot plots showing the average expression of selected genes among each cell type (including some marker genes) and the percentage of cells expressing the genes. (C) Split UMAP visualization of cells by individual samples. Cell coordinates are consistent with their positions in panel A. (D) Stacked bar plot of the cell type distribution among each dataset of the responder (R) and non‐responder PDAC samples (NR). (E) Box plot showing the % of specified cell types between chemotherapy responders and non‐responders. Labelled values represent *p‐*values from unpaired Wilcoxon‐rank sum test.

PDAC tissue specimens were collected from patients (*n* = 12) who underwent neoadjuvant chemotherapy (FOLFIRINOX (*n* = 9) and gemcitabine/nab‐paclitaxel (*n* = 3)) without radiation. Five of these patients (*n* = 5) responded to chemotherapy with a CAP histopathologic response score of 2 (partial response), and seven (*n* = 7) did not respond (CAP 3, no response). The demographics and clinical characteristics of the patients are reported in Table S1. Although we did not observe any statistically significant baseline differences between response groups, the cohort size was limited, and treatment‐regimen stratification was underpowered; therefore, regimen‐specific effects could not be reliably assessed. Single‐nuclei suspensions were generated from frozen tissue samples and sequenced using Illumina NextSeq 2000 sequencer. The preliminary aggregate cell count from the raw data was 48 557 cells. Following routine processing and quality assessment of the raw sequencing data, a total of 30 840 cells were retained for the study (Figure S1). To delineate the cellular composition and transcriptional heterogeneity across the PDAC tissue samples, we performed an unsupervised, graph‐based clustering and uniform manifold approximation and projection (UMAP) visualization, identifying thirteen distinct cell clusters, such as myeloid cells, endothelial cells, endocrine (α,β,δ) cells, T cells, B cells, fibroblasts, acinar cells, ductal cells, and mast cells, which were annotated using canonical markers and gene expression profiles (Figure 1A). We generated a violin plot showing the distinct expression of marker genes used to identify the various cell types (Figure S2A). Each cluster was validated by differential gene expression analysis, showing strong expression of representative markers referenced in the literature for identifying the various cell types (Figure 1B). The bubble plot displays both the average expression and percentage of key marker genes expressed, confirming the accuracy of our cell type annotation. We generated individual cell‐type counts in the overall dataset retained for analysis, and the percentage of cell type across the dataset was included using the Seurat project (Figures S2B,C). We next visualized cell types on a UMAP plot across individual responder and non‐responder samples (Figure 1C). Cells are tinted by response status only, with responders shown in blue (labelled ‘R’) and non‐responders in red (‘NR’). To investigate the various cell types and changes in cellular composition across individual samples, we quantified the percentage of each annotated cell type in each sample (Figure 1D). The stacked bar plot shows differences in the distribution of epithelial, stromal, and immune cells between responders and non‐responders. In addition, we presented the percentage of each cell type for both the responders and non‐responders using a stacked bar plot (Figure S2D). Furthermore, we compared the percentage of the various cell types between the responder and non‐responder groups using box plots (Figure 1E). Statistical comparisons using the unpaired Wilcoxon rank‐sum test suggest that myeloid cells showed the largest difference in abundance between responders and non‐responders (*p *= .13); however, this finding did not achieve statistical significance. This non‐significant trend, coupled with the pivotal function of myeloid cells in PDAC biology, prompted a focused exploratory analysis of the myeloid compartment. In summary, these findings delineate the cellular landscape of neoadjuvant‐treated PDAC and suggest response‐associated compositional differences, but the small sample size and variability preclude firm conclusions.

### 3.2 Gene set enrichment analysis highlights transcriptional differences in myeloid cells between responder and non‐responder PDAC samples

**FIGURE 2:**
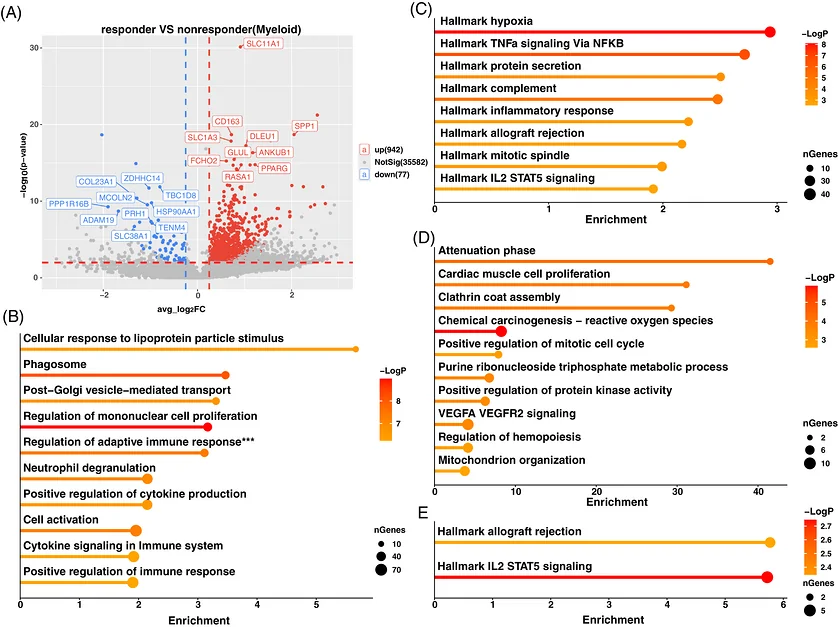
Differential gene expression and functional enrichment in myeloid cells from chemotherapy responder and non‐responder PDAC samples. (A) Volcano plot showing the upregulated (red) and downregulated (blue) genes in myeloid cells between PDAC chemotherapy responders and non‐responders. The *x*‐axis represents the log_2_ fold change, and the y‐axis shows the –log_10_ *p*‐value. Differentially expressed genes were defined as those with |log_2_ fold change| ≥ 0.25 and FDR (adjusted *p*‐value) ≤ .05. (B) Gene Ontology (GO) biological process enrichment of genes upregulated in responders. (C) Hallmark pathway enrichment for genes upregulated in responders. (D) Gene Ontology (GO) biological process enrichment of genes downregulated in responders. (E) Hallmark pathway enrichment for genes downregulated in responders. GO enrichment *p*‐value is indicated by bar colour with scale showing the –log_10_ *p*‐value to the right. These results are interpreted as exploratory due to the small cohort size and potential pseudoreplication; pathway enrichment analyses summarize broad transcriptional trends rather than establish definitive mechanistic roles for individual genes.

Myeloid cells are crucial to the progression of PDAC due to their immunomodulatory and stromal remodelling functions^27^; however, their intrinsic heterogeneity complicates efforts to identify subsets associated with chemotherapy response. To explore response‐associated transcriptional programs, we performed differential gene‐expression analysis of myeloid cells from responders versus non‐responders. We identified 942 genes upregulated and 77 downregulated. The volcano plot (Figure 2A) highlight genes (*SLC11A1*, *CD163*, *SPP1*) significantly upregulated in the responders and others downregulated (*ADAM19*, *COL23A1*) compared to non‐responders (FDR < .05, |log_2_FC| > .25). Notably, the upregulated genes are associated with tissue regeneration and immunosuppression.^50^, ^51^, ^52^ The *SPP1* gene, which showed the greatest fold‐change increase in expression, is a marker gene for TAMs involved in TME remodelling via intercellular communication.^53^ We next performed a gene set functional enrichment analysis using Metascape^43^ on the differentially expressed genes upregulated and downregulated in the PDAC responders, evaluating enrichment across pathway ontologies, hallmark gene sets and immunologic signatures. The top 5 enriched terms from each category were identified. The enriched pathway ontologies and hallmark gene sets for upregulated myeloid genes in the responders are shown in Figure 2B,C. The immunologic signatures specific to the immune pathways of the upregulated and downregulated myeloid genes in the responders are also shown (Figure S3A,B). The Gene Ontology (GO) in Figure 2B shows enrichment for biological processes involved in phagocytosis and neutrophil degranulation, suggesting active engulfment and processing of extracellular material by macrophages. The hallmark gene set further highlights gene sets related to hypoxia, TNF‐α signaling via NF‐κB, complement and inflammatory‐response pathways (Figure 2C). The Gene Ontology (GO) and hallmark gene set in the downregulated myeloid genes of PDAC chemotherapy responders suggest their association with a dysfunctional myeloid profile (Figure 2D,E). Because these analyses are based on cell‐level comparisons and a limited patient cohort, the enrichment findings are interpreted as candidate response‐associated programs rather than definitive patient‐level mechanisms.

Together, these results suggest that myeloid cells in responder tumours exhibit transcriptional programs consistent with phagocytosis, inflammatory signaling, complement activity and antigen‐processing functions. These results bolster the hypothesis that chemotherapy response in PDAC may involve functional remodelling of the myeloid compartment, but they do not establish that these programs are causal mediators of response.

### 3.3 Single‐nucleus transcriptomic analysis suggests differential myeloid cell composition and transcriptional signatures between responder and non‐responder PDAC samples

**FIGURE 3:**
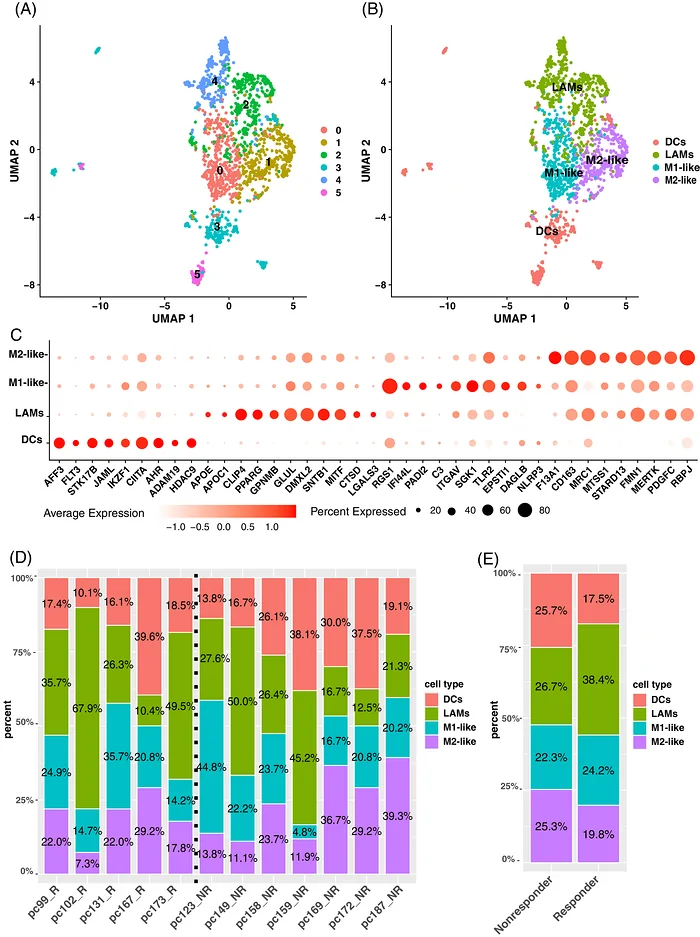
Transcriptomics analysis and signatures of the differential myeloid cell composition. (A) UMAP visualization of the clusters in the myeloid subsets. (B) UMAP representation of the annotated clusters with defined myeloid subsets. (C) Dot plot showing expression of key marker genes used to annotate each myeloid subset. Dot size reflects the fraction of cells expressing the gene, and colour indicates average expression. (D) Stacked bar plot showing the relative proportion of each myeloid subset per patient, highlighting inter‐patient variability and response‐associated composition patterns. R: responder; NR: nonresponder. (E) Boxplots comparing the percentage of each myeloid subset among the total myeloid cells between responders and non‐responders.

Due to myeloid cell heterogeneity, we investigated the composition of myeloid cell subpopulations in chemotherapy responders and non‐responders. We conducted subclustering analysis on the myeloid cell cluster from the snMultiome data. Unsupervised clustering at .5 resolution formed six myeloid subsets (Clusters 0–5) visualized using UMAP projection (Figure 3A). According to marker‐gene expression, these clusters were operationally annotated as lipid‐associated macrophages (LAMs), M1‐like macrophages, M2‐like macrophages and dendritic cells (Figure 3B). The heatmap in Figure S4 shows the representative genes used to identify myeloid subsets, corroborated by marker genes identified in the literature, as referenced below. The expression of the top significant marker genes used for identification of the myeloid subcluster is shown using a dot plot displaying the average expression of the marker genes and the percentage of genes expressed (Figure 3C). The M1‐like and M2‐like terms are used here as practical descriptors of transcriptional states rather than fixed polarization identities, recognizing that tumour macrophage states exist along continua and may share overlapping programs. The dendritic cells (DC) were identified with representative markers such as *ADAM19*,^54^ *FLT3* ^55^ and *AFF3*. M1‐like macrophages were identified by the markers *RGS1*, *SGK1*, *ITGAV* and *PADI2*, while the M2‐like macrophages were characterized by *CD163*,^27^ *MRC1*,^56^ *FMN1* and *RBPJ*. A subset was identified as lipid‐associated macrophages (LAMs) using *APOE, APOC1*,^19^ *GPNMB*,^34^ *CLIP4*, DMXL2 and *PPARG*, which play an essential role in lipid synthesis,^57^ and *CTSB, LGALS3* and *LGALS1*, which are associated with lipid metabolism and phagocytosis.^58^ To further evaluate inter‐patient variability in myeloid subset distribution, we quantified the percentage of each subcluster across individual samples (Figure 3D). LAMs are tumour‐infiltrating, monocyte‐derived macrophages characterized by a lipid‐laden phenotype and transcriptional program that regulates inflammation, tissue remodelling, and metabolic stress. Notably, the composition of myeloid cells varied considerably between patients, with certain clusters enriched in chemotherapy responders, suggesting patient‐specific immune remodeling. In addition, we clearly demonstrated the overall percentage of myeloid subsets in responders and non‐responders using a box plot to compare the distribution of response types. We observed that LAMs accounted for a higher percentage of the myeloid compartment in responders (38.4%) than in non‐responders (26.7%), although this difference is a trend in a small cohort (Figure 3E). Taken together, these results suggest a transcriptionally and compositionally distinct myeloid landscape in PDAC based on treatment response, characterized by a trend toward a high percentage of LAMs among myeloid cells in responders. M1‐like macrophages also tended to be more abundant in responders, whereas non‐responders tended to have relatively higher fractions of M2‐like macrophages and dendritic cells. These results suggest response‐associated compositional variation within the myeloid compartment and support the hypothesis that chemotherapy response in PDAC may be associated with a bias toward inflammatory and lipid‐handling myeloid programs; nonetheless, due to the limited sample size and lack of statistical significance, these associations are considered exploratory.

### 3.4 Gene set functional enrichment analysis reports the functional states of the myeloid subsets between responder and non‐responder PDAC samples

**FIGURE 4:**
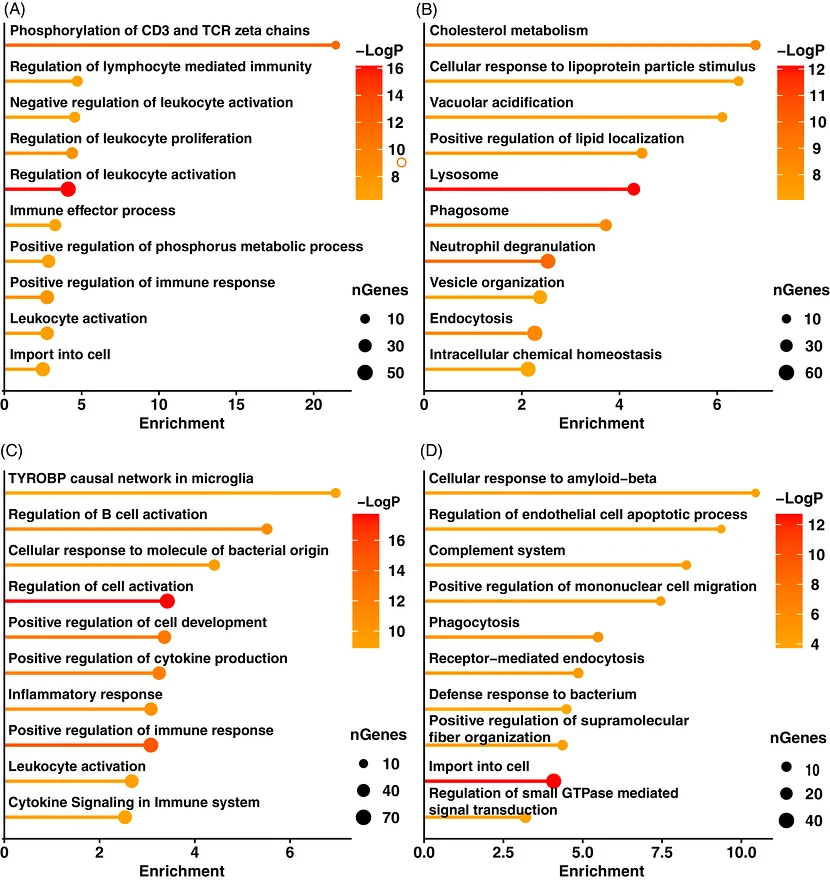
Gene Ontology enrichment defines functional programs of the myeloid clusters. (A) Gene Ontology biological processes of dendritic cells (DCs). (B) Gene Ontology biological processes of lipid‐associated macrophages (LAMs). (C) Gene Ontology biological processes of the classically activated macrophages (M1 macrophages). (D) Gene Ontology biological processes of the alternatively activated macrophages (M2 macrophages). DCs show enrichment for leukocyte activation, T‐cell receptor signaling, and adaptive immune response, consistent with immunogenic antigen‐presenting functions. LAM‐like macrophages are enriched for lysosomal, phagocytic, and endocytic pathways, indicating active uptake and processing of extracellular material and lipids. M1‐like macrophages are associated with pro‐inflammatory and cytokine‐regulatory pathways, whereas M2‐like macrophages show enrichment for wound healing, tissue repair, angiogenesis, and extracellular matrix remodelling. These functional annotations support the interpretation of transcriptionally defined myeloid states but are presented as hypothesis‐generating rather than definitive mechanistic assignments.

To clarify the functional traits of transcriptionally distinct myeloid populations, we conducted Gene Ontology (GO) analyses on cluster‐specific differentially expressed genes to identify the unique attributes of these myeloid subsets (Figure 4A–D). The GO biological process in the dendritic cell cluster Figure 4A (top left) shows a significant upregulation of genes associated with leukocyte activation, T cell receptor signaling, and adaptive immune response, consistent with antigen‐processing and immune‐interaction programs. LAM‐like macrophages showed enrichment of lysosome‐related pathways, neutrophil degranulation, phagocytosis, and endocytosis, consistent with uptake and processing of extracellular material and cellular debris^59^, ^60^, ^61^ (Figure 4B). M1‐like macrophage, regarded as classically activated, showed pathways related to pro‐inflammatory signaling and regulation of cytokine responses, which can further contribute to an anti‐tumour response (Figure 4C), whereas M2‐like macrophages showed pathways related to wound healing, tissue repair, angiogenesis, and phagocytosis (Figure 4D), reinforcing the characteristics of alternatively‐activated macrophages (M2 macrophages),^62^ a phenotype often associated with tumour progression, metastasis, and unfavourable prognosis in PDAC^63^, ^64^ including many other tumour types. These enrichment analyses suggest that myeloid subpopulations in PDAC possess distinct functional programs, encompassing lipid handling, phagocytosis, antigen processing, inflammatory signalling and tissue remodeling. Given the exploratory nature of these analyses, we regard them as indicative of functional heterogeneity rather than conclusive evidence that any specific subset directly mediates chemotherapy response.

### 3.5 Pseudotime trajectory analysis of the myeloid subset from the scRNA‐Seq dataset

**FIGURE 5:**
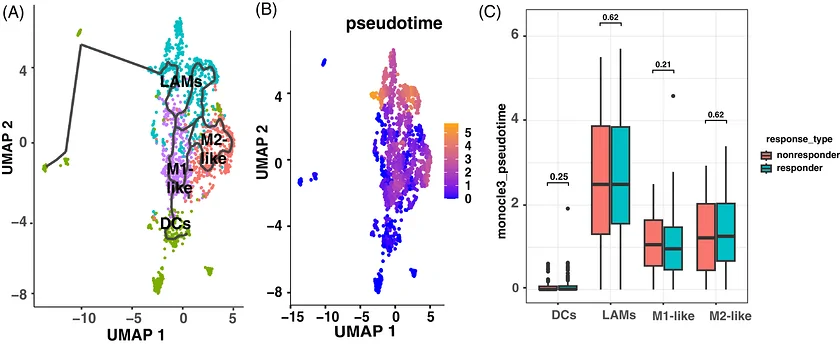
Inferred pseudotime continuum of myeloid states in neoadjuvant‐treated PDAC. (A) UMAP projection of the myeloid subset coloured by pseudotime values inferred using a trajectory analysis framework. (B) Pseudotime projection on UMAP. (C) Boxplot visualization of pseudotime distributions across myeloid subsets between chemotherapy responders and non‐responders. We oriented the trajectory so that dendritic cells occupy the earliest pseudotime values, based on established biological knowledge. Altogether, this result is interpreted as an inferred transcriptional continuum and does not establish a definitive developmental lineage from DCs to LAMs.

Pseudotime trajectory analysis can provide insight into the transcriptional continua among related cell states, but it does not by itself establish lineage relationships. Macrophages are heterogeneous and can transdifferentiate between phenotypes in response to environmental signals.^12^ Using the trajectory inference algorithm, we constructed an inferred transcriptional continuum of myeloid cells, visualized on the UMAP (Figure 5A,B). Mapping cells along pseudotime suggested a progression from DC‐enriched regions with low pseudotime values toward macrophage states, including M1‐like, M2‐like, and LAM‐like populations, at higher pseudotime values. In our analysis, we oriented the trajectory such that dendritic cells were specified as the root based on established biological knowledge, and all other cells were assigned relative positions along the inferred trajectory. Therefore, our analysis does not establish a definitive developmental lineage from DCs to macrophages (Figure 5B). The trajectory supports a directional path originating from dendritic cells (DCs), which could be monocyte‐derived dendritic cells^65^, ^66^ further differentiating into the M1‐like macrophages, M2‐like macrophages, and terminal state enriched for lipid‐associated macrophages (LAMs). Because the root was assigned based on biological interpretation and observed pseudotime structure, rather than direct lineage tracing, RNA velocity, or fate‐probability modelling, this analysis is viewed as a putative transcriptional‐state continuum. To quantitatively assess pseudotime distribution across annotated subtypes, we compared the distribution of pseudotime values across DCs, M1‐like, M2‐like and LAM subsets between chemotherapy responders and non‐responders using the Wilcoxon rank‐sum test, and there was no statistically significant difference in pseudotime observed between the response groups, suggesting that response‐associated differences in the myeloid compartment are likely attributed to subset abundance and transcription or regulatory programs rather than an overall shift in pseudotime state (Figure 5C). Therefore, this data is interpreted as exploratory rather than evidence of response‐specific lineage progression.

Together, these data suggest an inferred continuum of tumour‐infiltrating myeloid states in PDAC, with LAMs occupying the later region of pseudotime in this analysis. However, pseudotime values did not differ significantly between responders and non‐responders within individual myeloid subsets, indicating that response‐associated myeloid differences are not explained by an overall shift in pseudotime state. These results support the interpretation of LAM‐like macrophages as a transcriptionally remodelled myeloid state but do not establish a definitive lineage trajectory. Future studies incorporating RNA velocity or experimental fate mapping will be required to determine whether LAM‐like macrophages arise through progressive differentiation, local reprogramming, selective recruitment, or expansion of pre‐existing macrophage states.

### 3.6 ChromVAR analysis identifies myeloid subset and response‐associated regulatory motif programs in PDAC

**FIGURE 6:**
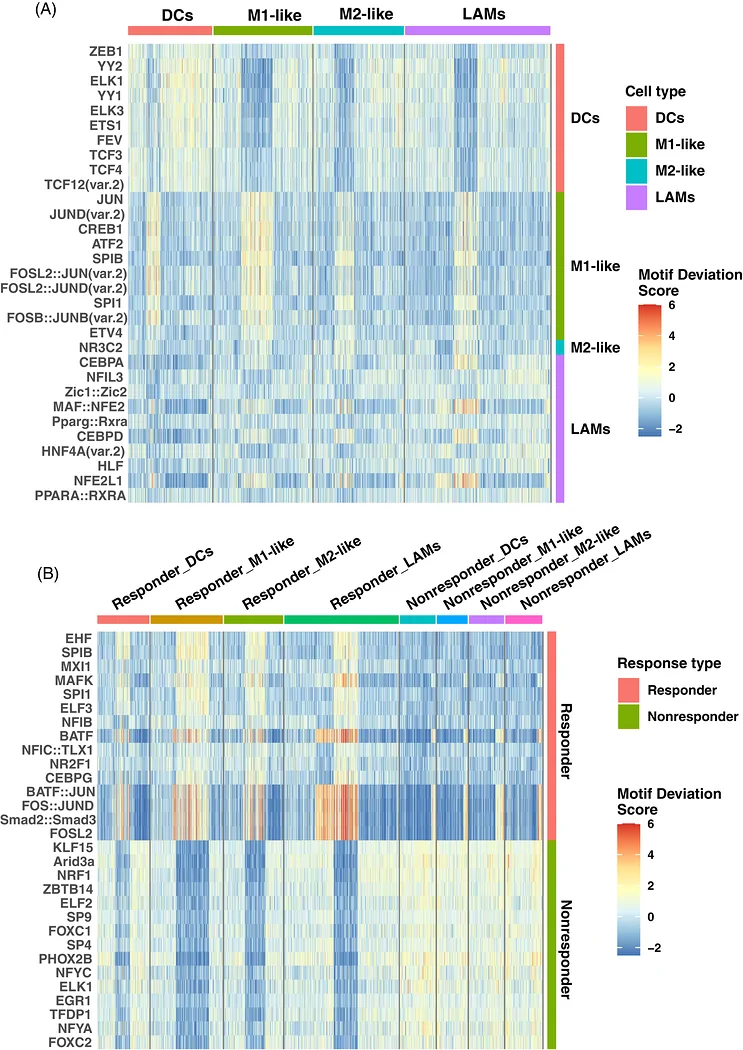
Chromatin accessibility and transcriptional‐factor motif patterns across myeloid subsets and response groups. (A) Heatmap displaying ChromVAR deviation scores for individual cells. Each row represents a motif, and each column represents a single cell. For each cell type, the top 10 cell type‐specific motifs were selected based on the adjusted *p* value (p_val_adj). Only one motif in the M2 cell type met the significance threshold. (B) Heatmap displaying ChromVAR deviation scores for each cell type across responder and non‐responder. Each row represents a motif, and each column represents a single cell across response type. The top 15 motifs associated with each response type were selected from the chromVAR marker analysis and ranked by adjusted *p* value (p_val_adj). Motif analyses are based on chromVAR deviation scores averaged across cells in each group and are presented descriptively. Because we did not perform patient‐level hypothesis testing for individual motifs in this small cohort, these patterns highlight candidate regulatory programs that may underlie myeloid state differences but should not be over‐interpreted as definitive mechanistic drivers of chemotherapy response.

To investigate candidate regulatory programs underlying myeloid cell states in neoadjuvant chemotherapy‐treated PDAC, we analysed the ATAC‐seq component of the snMultiome dataset using ChromVAR.^42^ ChromVAR uses accessible ATAC regions as input to assess the enrichment of transcription factor (TF) motifs from the JASPAR database.^67^, ^68^ It also calculates a transcription factor motif deviation score for each cell, indicating how accessible a motif is relative to the background. A positive deviation score means the motif accessibility is higher than the background, while a negative deviation score means the motif accessibility is lower. In our analysis, we first examined cell type‐specific motif‐accessibility patterns across the annotated myeloid subsets, including dendritic cells (DCs), M1‐like macrophages, M2‐like macrophages, and lipid‐associated macrophages (LAMs) (Figure 6A). For each myeloid subset, the top 10 cell type‐specific motifs were selected based on the adjusted *p* value (p_val_adj) and visualized as a heatmap of individual cell chromVAR deviation scores (Figure 6A; Supporting Information). Each row represents one transcription factor binding motif. Only one motif in the M2 cell type met the significance threshold, so we included one motif in the heatmap (Figure 6A). In this analysis, motifs such as Erythroblast Transformation‐Specific 1 (ETS1), T Cell Factor (TCF3, TCF4 and TCF12), Yin Yang 1 and 2 (YY1, YY2) in the DC suggest candidate regulatory programs involved in DC activation and immune‐cell state regulation.^69^, ^70^, ^71^ Motifs in the M1‐like macrophage group consist of the Activator Protein 1 (AP‐1)^72^ family, such as JUN, JUND, FOSL2::JUND, FOSL2::JUN, FOSB::JUNB, cAMP‐responsive element binding protein 1 (CREB1),^73^ which are commonly associated with inflammatory and stress‐response and macrophage activation programs.^74^ The LAMs displayed motifs such as nuclear factor erythroid 2‐related factor 1 (NFE2L1),^75^ MAF:NFE2,^76^ CCAAT/enhancer binding protein alpha and delta (CEBPA and CEBPD),^77^, ^78^ and peroxisome proliferator‐activated receptor (PPAR)^79^ family (PPARA::RXRA, Pparg::Rxra), which are associated with lipid metabolism, lipid sensing/inhibition of lipid accumulation, and macrophage differentiation. We next assessed differences in motif‐accessibility patterns by chemotherapy response status by stratifying myeloid cells into response groups and subset identities (Figure 6B). To explore response‐associated differences, the top 15 motifs for each response type were selected from the chromVAR marker analysis and ranked by adjusted *p*‐value (p_val_adj) (Figure 6B; Supporting Information). In this analysis, we observed an apparent difference in motif accessibility between responder and non‐responder myeloid populations. Responder‐associated myeloid cells, particularly responder LAM‐like macrophages, showed higher accessibility of activator protein (AP‐1)/basic leucine zipper ATF‐like Transcription Factor (BATF)/SMAD‐related motifs, including FOSL2, FOS::JUND, BATF::JUN, BATF, and Smad2::Smad3. These motifs suggest candidate regulatory programs linked to macrophage activation, stress‐response signaling, inflammatory remodeling, and tissue remodeling.^80^, ^81^ In contrast, the non‐responder‐associated myeloid cells showed increased accessibility of a distinct set, such as ETS‐domain DNA‐binding sequence (ELK1), Forkhead box C1 and C2 (FOXC1, FOXC2), A‐T rich interacting domain 3a (Arid3a), ELF2, and nuclear transcription factor Y subunit gamma (NFYC) motifs linked to stress response, tumour progression, MAPK‐associated signaling, chromatin remodeling and metabolic adaptation.^82^, ^83^, ^84^ These findings nominate transcription factor groups that may contribute to response‐associated myeloid states; however, chromVAR alone cannot determine whether these factors are functionally active or causally responsible for the observed phenotypes.

Together, our ATAC‐Seq analyses suggest that the myeloid subsets exhibit distinct chromatin accessibility landscapes and that some motif‐accessibility patterns vary by response group. These data are consistent with candidate regulatory differences involving inflammatory, myeloid lineage, and immunometabolic programs. However, given the small cohort size and descriptive nature of ChromVAR motif scoring, we avoid concluding that coordinated epigenetic reprogramming drives chemotherapy response. Instead, these results identify regulatory hypotheses that require validation by larger datasets, orthogonal assays, and functional perturbation.

### 3.7 Spatial transcriptomics provides a descriptive comparison of macrophage‐predominant lipid‐associated macrophages (LAMs) in chemotherapy‐treated and untreated PDAC samples

**FIGURE 7:**
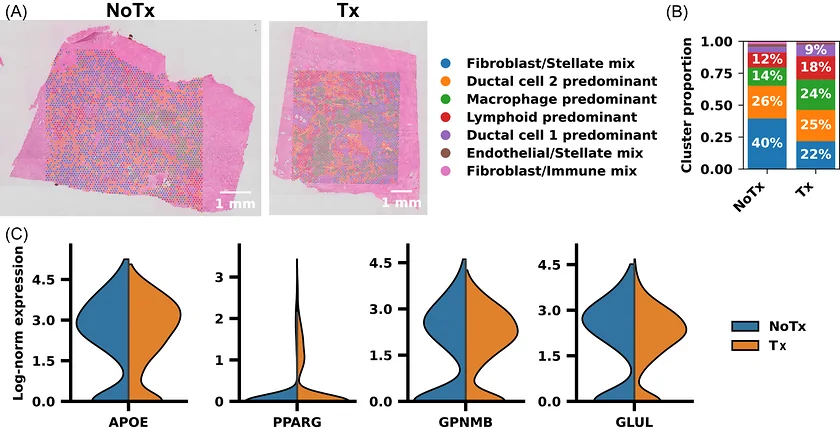
Descriptive spatial transcriptomic comparison of LAMs in PDAC chemotherapy‐treated responder and untreated samples. (A) Spatial visualization of cluster annotations for PDAC+NoTx (left) and PDAC+Tx (right). (B) Stacked bar plot showing cluster proportions for PDAC+NoTx (left) and PDAC+Tx (right). (C) Violin plots showing the expression of LAM marker genes in the macrophage‐predominant cluster for PDAC+NoTx (left/blue) and PDAC+Tx (right/orange). This spatial analysis compares one chemotherapy‐treated responder with one untreated PDAC specimen, thereby representing a distinct biological contrast from the main single‐nucleus comparison of chemotherapy‐treated responders versus chemotherapy‐treated non‐responders. The spatial data are intended as descriptive, cross‐platform, hypothesis‐generating support and should not be viewed as strong independent validation of responder‐specific myeloid remodelling.

In recent years, integrating spatial transcriptomics with complementary omics technologies has yielded an unparalleled understanding of the spatial arrangement and molecular heterogeneity of cells within tissues. This integration addresses the spatial information loss often associated with scRNA‐seq and holds great potential for applications in biology.^85^ Spatial transcriptomics can contextualize transcriptomic programs within tissue architecture, but the spatial component of this study was limited to one chemotherapy‐treated PDAC tumour (PDAC+Tx; responder) and one untreated PDAC tumour (PDAC+NoTx). Therefore, this analysis does not directly validate the primary responder‐versus‐non‐responder comparison from the snMultiome cohort. Rather, it provides a descriptive cross‐platform comparison of macrophage‐predominant regions and LAM‐associated marker expression between a chemo‐treated responder and untreated tissue. Figure 7A shows the H&E‐stained spatial transcriptomic sections from the PDAC+NoTx sample (left) and the PDAC+Tx sample (right), with capture spots color‐coded by annotated clusters. In this pairwise comparison, the chemotherapy‐treated responder sample appeared to contain a larger macrophage‐predominant region than the untreated sample. The untreated sample contained a macrophage‐predominant region comprising 14% of QC‐retained, tissue‐associated spots, whereas the chemotherapy‐treated responder sample showed a macrophage‐predominant region comprising 24% of QC‐retained, tissue‐associated spots (Figure 7B). We then compared expression of LAM‐associated genes in macrophage‐predominant regions between the chemotherapy‐treated responder and the untreated sample using violin plots (Figure 7C). LAM‐associated markers appeared higher in the treated responder sample than in the untreated sample. These results are descriptive and hypothesis‐generating and may reflect treatment exposure, response status, patient‐specific biology, or tissue‐region differences.

In summary, the spatial transcriptomics data suggest that macrophage‐predominant regions and LAM‐associated marker expression can be visualized within the PDAC tissue context. However, because the analysis compares only one treated responder with one untreated tumour, it is not presented as independent validation of LAM enrichment in responders versus non‐responders. Larger spatial cohorts, ideally including matched pre‐treatment and post‐treatment specimens from responders and non‐responders, will be required to determine whether LAM‐like macrophage regions are reproducibly associated with chemotherapy response.

## 4 DISCUSSION AND CONCLUSION

Pancreatic ductal adenocarcinoma (PDAC) continues to be one of the most lethal human cancers, and most patients derive limited durable benefit from current systemic therapies. While only 20% of patients are eligible for surgical resection, chemotherapy remains the cornerstone of systemic therapy. Regimens such as FOLFIRINOX or gemcitabine combined with nab‐paclitaxel have significantly improved long‐term outcomes and survival rates for PDAC patients.^86^, ^87^, ^88^ However, intrinsic and acquired resistance to chemotherapy remains a major driver of treatment failure in PDAC. The PDAC tumour microenvironment contributes to therapy resistance through stromal, metabolic, and immune‐suppressive mechanisms, and much attention has been paid to targeting the diverse compartments of the TME or combining it with other treatment modalities.^89^, ^90^ Myeloid cells are key regulators of the immune‐suppressive characteristics of the PDAC TME.^91^ They exhibit functional plasticity and can exert either pro‐tumorigenic or anti‐tumour effects depending on their phenotypic state and environmental cues.^12^ Hence, understanding how chemotherapy modulates the myeloid compartment in PDAC may inform the development of predictive biomarkers and rational combinatorial strategies to overcome therapeutic resistance. Previous studies have used single‐cell/nucleus RNA sequencing, spatial transcriptomics, and bulk proteogenomics to investigate tumour heterogeneity, cellular subpopulations and treatment responses in PDAC.^92^ Other studies have reported the transcriptomic landscape in PDAC and the effects of chemotherapy on the human PDAC TME using single‐cell RNA sequencing.^29^ To expand the body of scientific knowledge, and to address a gap in the field, we used integrated single‐nucleus transcriptomic and chromatin‐accessibility profiling in this exploratory study to examine myeloid states associated with histopathologic response to neoadjuvant chemotherapy in PDAC. Rather than establishing a definitive mechanism, our data nominate response‐associated myeloid programs, including LAM‐like macrophage states, for further validation.

A key observation motivating this study is a numerical difference in myeloid abundance between responders and non‐responders that did not reach statistical significance. Although myeloid cells showed the largest difference in abundance among major cell lineages, with a *p*‐value of .13, it is not considered a robust group‐level effect. Therefore, we used this trend as a starting point to focus on myeloid heterogeneity rather than as a definitive finding.  Using single‐nucleus multiomics, we further identified heterogeneous myeloid cells in neoadjuvant‐treated PDAC tumours from histopathologic responders and non‐responders. Within the myeloid compartment, subclustering identified dendritic cells, M1‐like macrophages, M2‐like macrophages, and a LAM‐like subset defined by genes involved in lipid handling, scavenger function, and phagocytosis. Responders tended to exhibit higher proportions of LAM (38.4%) (compared to non‐responders, 26.7%), and M1‐like populations, whereas non‐responders tended to harbour relatively higher fractions of M2‐like and dendritic cells. However, these compositional differences also did not consistently reach conventional significance thresholds, and substantial inter‐patient variability was present. LAMs are a subset of the tumour macrophages^51^ characterized by their lipid‐handling capacity and expression of genes such as *TREM2*, *APOE, CTSL* and *GPNMB* that can switch phenotype based on environmental cues.^12^, ^33^, ^58^ In this study, we therefore frame LAM enrichment and related myeloid shifts as trends that suggest association with response rather than as statistically proven discriminators between clinical groups.

Several non‐mutually exclusive explanations may account for the observed trend toward higher proportions of LAM‐like macrophages in responders, and each warrants further testing. First, chemotherapy‐induced tumour cell death may increase apoptotic debris, oxidized lipids, and damage‐associated molecular patterns (DAMPs) that promote recruitment or local remodeling of lipid‐scavenging macrophages that engage in lipid uptake and efferocytosis. Second, chemotherapy‐associated hypoxia and oxidative stress may induce programs that promote lipid uptake, lysosomal biogenesis, and metabolic reprogramming in macrophages, thereby driving LAM identity. Third, TAMs can present both classically activated (M1) and alternatively activated (M2) characteristics.^93^ M1‐like macrophages are associated with high glycolytic metabolism and a robust ability to generate reactive oxygen species (ROS), supporting their pro‐inflammatory and cytotoxic functions.^94^ M2‐like macrophages use oxidative phosphorylation as their main energy source, facilitating their tissue repair ability.^95^ The metabolic profile of TAMs is dynamic^96^; therefore, LAM‐like macrophages in treated PDAC may represent a context‐specific macrophage state that differs from LAM populations described as tumour‐promoting or immunosuppressive in other cancers. Our findings do not imply that all LAMs are beneficial, but rather that LAM‐like programs in chemotherapy‐treated PDAC may reflect therapy‐associated tissue remodelling, altered phagocytic demand, or a pre‐existing myeloid state associated with responsiveness. Because all snMultiome specimens were collected after treatment and matched baseline biopsies were unavailable, we cannot determine whether LAM‐like enrichment preceded chemotherapy, was induced by treatment, or arose from both processes.

Transcriptional profiling of myeloid cells revealed response‐associated differences in gene expression and pathway enrichment that align with established myeloid functions in PDAC. In responders, myeloid cells upregulated gene programs related to phagocytosis, antigen processing and presentation, cytokine signaling and inflammatory remodeling. These included pathways associated with complement activation, TNF‐α signaling, and hypoxia‐driven responses, consistent with a more immune‐activated myeloid compartment. In contrast, genes downregulated in responder myeloid cells were enriched for processes suggestive of dysfunctional or suppressive states. Taken together, these patterns support the hypothesis that chemotherapy responsiveness may be associated with a myeloid compartment biased toward phagocytic, inflammatory, and antigen‐presenting programs, potentially favouring immune‐mediated tumour control. At the same time, differential expression analyses were performed largely at the cell level with modest sample numbers, and although we applied multiple‐testing correction and, where feasible, patient‐level aggregation, the risk of pseudoreplication and overestimation of significance remains. For this reason, we view specific gene‐level findings as provisional and emphasize pathway‐level trends rather than individual markers as mechanistic endpoints.

Our ATAC‐seq analysis extends the transcriptomic findings by suggesting that PDAC‐infiltrating myeloid subsets occupy distinct candidate regulatory states. The LAMs exhibited motif patterns associated with lipid/metabolic regulation, stress‐response mechanisms, and macrophage differentiation, supporting their annotation as a distinct macrophage state within the neoadjuvant chemotherapy‐treated PDAC tumour microenvironment.^58^, ^97^ When categorized by chemotherapy response, responder‐associated LAMs showed increased accessibility of AP‐1/BATF/SMAD‐related motifs, suggesting candidate regulatory programs implicated in macrophage activation, inflammatory remodeling, stress‐response signalling, and tissue remodelling. These patterns are consistent with the responder‐associated transcriptional enrichment of phagocytosis, antigen processing, complement activity, inflammatory signalling and lipid‐handling programs. Conversely, non‐responder‐associated myeloid cells showed significant motif patterns, but with a distinct motif set, including ELK1, FOXC1, FOXC2, Arid3a, ELF2 and NFYC, suggesting that non‐responder myeloid cells may occupy an alternative regulatory state characterized by cell‐state regulation, chromatin‐associated transcriptional control and metabolic adaptation. The patterns from our ChromVAR analysis are biologically plausible given the roles of these transcription factor families in macrophage activation, myeloid differentiation, inflammatory signalling, and metabolic adaptation. However, ChromVAR measures motif‐associated chromatin accessibility rather than direct transcription factor activity, and the current analysis was based on averaged motif‐deviation scores from a small cohort. Therefore, these findings are interpreted as candidate regulatory signatures, not proof that epigenetic reprogramming drives chemotherapy response. Future studies would incorporate patient‐level statistical modelling, effect sizes, adjusted significance testing of motif differences and orthogonal validation of candidate transcription factors.

To complement single nucleus multiomics, we performed spatial transcriptomic profiling in one chemotherapy‐treated PDAC responder and one untreated PDAC tumour. In this descriptive cross‐platform comparison, macrophage‐rich regions expressing LAM‐associated genes appeared more prominent in the treated specimen. These spatial patterns are consistent with the notion that chemotherapy exposure can be associated with enrichment of LAM‐like programs in the myeloid compartment. However, this comparison involves only two patients and contrasts a treated responder with an untreated tumour rather than directly comparing treated responders and treated non‐responders. As such, the spatial analysis is interpreted as illustrative and hypothesis‐generating, not as an independent validation of the responder‐versus‐non‐responder multiome findings. Future spatial studies in larger, treatment‐annotated cohorts will be needed to determine whether LAM‐like spatial niches reproducibly associate with chemotherapy response in PDAC.

We also explored the relationship between LAM‐associated transcriptional signatures and overall survival in the Pancreatic Cancer Action Network's (PanCAN) Know Your Tumor® (KYT) cohort from the PanCAN SPARK Platform (Supporting Information (Method)).^98^ In the dataset, a higher LAM‐signature score was associated with improved survival only when patients were dichotomized by the median signature expression (Figure S5), whereas modelling the signature as a continuous variable did not yield a significant association. This pattern is statistically less robust and may be sensitive to the choice of threshold. In addition, treatment regimens and response information from the external cohort are heterogeneous and only partially aligned with our neoadjuvant chemotherapy setting. Therefore, we present the external survival analysis as exploratory support for a potential link between LAM‐like programs and clinical outcome, but not as evidence that LAM signatures are validated prognostic biomarkers. Larger, treatment‐stratified cohorts with harmonized clinical data will be required to rigorously assess the prognostic and predictive value of LAM‐associated signatures.

This study has several limitations. First, the cohort size is small (*n* = 12), with only five CAP 2 responders and seven CAP 3 non‐responders, which limits statistical power. Many observed differences, including those in myeloid abundance and LAM proportions, did not reach conventional significance thresholds and display substantial inter‐patient variability. Second, patients received heterogeneous neoadjuvant chemotherapy regimens (FOLFIRINOX, gemcitabine/nab‐paclitaxel, or both), and treatment duration varied. The sample size is underpowered for robust regimen‐stratified analyses, and we cannot rule out the possibility that the myeloid features we observe largely reflect regimen choice, treatment intensity, baseline disease biology, or other unmeasured confounders rather than response status per se. Third, all multiomic samples were collected at the time of surgical resection after treatment, without matched pre‐treatment biopsies. As a result, we cannot distinguish whether LAM‐like enrichment and other myeloid changes were preexisting features of tumors that subsequently responded to chemotherapy or were induced or expanded by chemotherapy itself. Fourth, because this study was designed and powered primarily to interrogate myeloid heterogeneity, we did not perform formal systematic analyses of T‐cell functional states, immune checkpoint status, or cancer‐associated fibroblast subtypes. Therefore, the data generated for this study are insufficient to define how myeloid remodeling affects T cell and stromal dynamics in PDAC. Future work using larger cohorts, including our ongoing mouse studies, will incorporate information on how myeloid remodeling/LAM‐like macrophage states in the PDAC TME, in the context of chemotherapy, affect T‐cell activation/exhaustion and CAF heterogeneity. Fifth, we did not perform orthogonal experimental validation of LAM markers in an independent cohort, so we did not confirm if APOE, APOC1, GPNMB, and TREM2‐expressing macrophages are reproducibly enriched in responders at the protein level. Therefore, future studies will validate these markers using flow cytometry or immunofluorescence in a PDAC murine model or a larger cohort of human samples. Finally, the multiomic analyses presented here are largely correlative. We did not perform functional perturbation experiments to test whether LAM‐like or other myeloid states causally modulate chemotherapy response; additional gene knockout, depletion,^35^ or reprogramming studies in relevant model systems will be essential to determine whether specific myeloid programs are necessary or sufficient for therapy sensitivity. Based on the hypothesis generated by this study, we are working to conduct mechanistic experiments using murine PDAC models.

Despite these limitations, our study provides a multi‐layered, human‐based view of myeloid diversity in neoadjuvant‐treated PDAC and generates testable hypotheses. The data suggest that chemotherapy response may be associated with a myeloid landscape enriched for LAM‐like and inflammatory macrophage programs, coupled with distinct chromatin accessibility patterns and spatial organization, whereas non‐response is associated with myeloid states biased toward tissue repair and angiogenesis. These observations highlight myeloid immunometabolism and LAM‐like programs as promising areas for mechanistic investigation and biomarker development in PDAC. Future work would prioritize larger, multi‐institutional cohorts with uniform treatment and response annotation, the incorporation of pre‐ and post‐treatment samples, rigorous patient‐level statistical modelling, and orthogonal functional studies to determine whether and how specific myeloid states can be leveraged to improve chemotherapy outcomes in this challenging disease.

## AUTHOR CONTRIBUTIONS

*Conceptualization*: Mercy Ojetunde, Brian A. Boone and Timothy D. Eubank. *Methodology*: Mercy Ojetunde, Hillary G. Goodney, Alyson M. Stevens, Alan Mizener and Li Ma. *Assays*: Mercy Ojetunde, Hillary G. Goodney, and Alyson M. Stevens. *Data analysis*: Li Ma, Mercy Ojetunde, Alan Mizener, Gangqing Hu, Barbara Szomolay, Kawther Abdilleh, Sascha Ott and Emidio E. Pistilli. *Writing*: Mercy Ojetunde and Li Ma. *Supervision*: Brian A. Boone, Timothy D. Eubank and Gangqing Hu.

## CONFLICT OF INTEREST STATEMENT

The authors declare no conflicts of interest.

## ETHICS STATEMENT

This study was reviewed and approved by the Institutional Review Board of West Virginia University (#1903496995), and written informed consent was obtained from all participants.

## Supporting information




**Table S1**: ctm270810‐sup‐0001‐TableS1.xlsx

**Table S2**: ctm270810‐sup‐0002‐TableS2.xlsx

**Table S3**: ctm270810‐sup‐0003‐TableS3.xlsx

**Table S4**: ctm270810‐sup‐0004‐TableS4.xlsx

**Figure S1**: ctm270810‐sup‐0005‐FigureS1.pdf

**Figure S2**: ctm270810‐sup‐0006‐FigureS2.pdf

**Figure S3**: ctm270810‐sup‐0007‐FigureS3.pdf

**Figure S4**: ctm270810‐sup‐0008‐FigureS4.pdf

**Figure S5**: ctm270810‐sup‐0009‐FigureS5.pdf

**Supporting File**: ctm270810‐sup‐0010‐FigureLegends.docx

## Data Availability

All raw sequencing data of snMultiome are publicly available at Gene Expression Omnibus under accession number GSE300595. All raw sequencing data of the spatial transcriptomics data are publicly available at Gene Expression Omnibus under accession number GSE337859.
