# Supplementary material for: Exploratory single‐nucleus multiomics analysis of myeloid cell states associated with neoadjuvant chemotherapy response in pancreatic ductal adenocarcinoma
Source: Clin Transl Med. 2026 Sep 14;16(9):e70810. doi: 10.1002/ctm2.70810 (PMC13574543; doi:10.1002/ctm2.70810)
Supplement: Supplementary file 1 [file CTM2-16-e70810-s001.pdf]

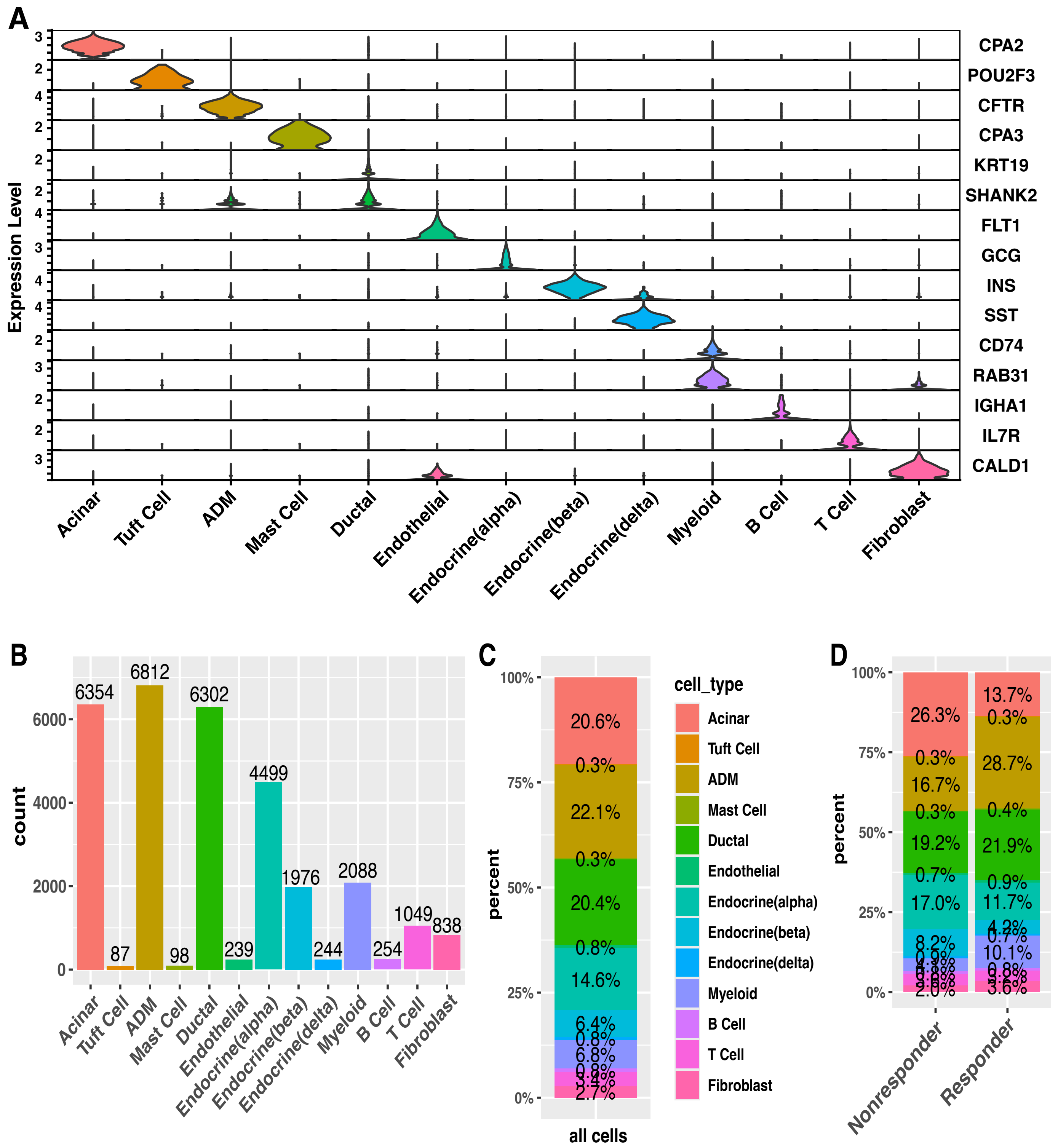

A
Expression Level
CPA2
POU2F3
CFTR
CPA3
KRT19
SHANK2
FLT1
GCG
INS
SST
CD74
RAB31
IGHA1
IL7R
CALD1
Acinar
Tuft Cell
ADM
Mast Cell
Ductal
Endothelial
Endocrine(alpha)
Endocrine(beta)
Endocrine(delta)
Myeloid
B Cell
T Cell
Fibroblast
B
count
6354
87
6812
98
6302
239
4499
1976
244
2088
254
1049
838
C
percent
all cells
20.6%
0.3%
22.1%
0.3%
20.4%
0.8%
14.6%
6.4%
0.8%
6.8%
3.4%
2.7%
cell_type
Acinar
Tuft Cell
ADM
Mast Cell
Ductal
Endothelial
Endocrine(alpha)
Endocrine(beta)
Endocrine(delta)
Myeloid
B Cell
T Cell
Fibroblast
D
percent
26.3%
0.3%
16.7%
0.3%
19.2%
0.7%
17.0%
8.2%
2.0%
13.7%
0.3%
28.7%
0.4%
21.9%
0.9%
11.7%
4.2%
0.7%
10.1%
0.8%
3.6%
Nonresponder
Responder
