# Supplementary material for: Exploratory single‐nucleus multiomics analysis of myeloid cell states associated with neoadjuvant chemotherapy response in pancreatic ductal adenocarcinoma
Source: Clin Transl Med. 2026 Sep 14;16(9):e70810. doi: 10.1002/ctm2.70810 (PMC13574543; doi:10.1002/ctm2.70810)
Supplement: Supplementary file 2 [file CTM2-16-e70810-s002.pdf]

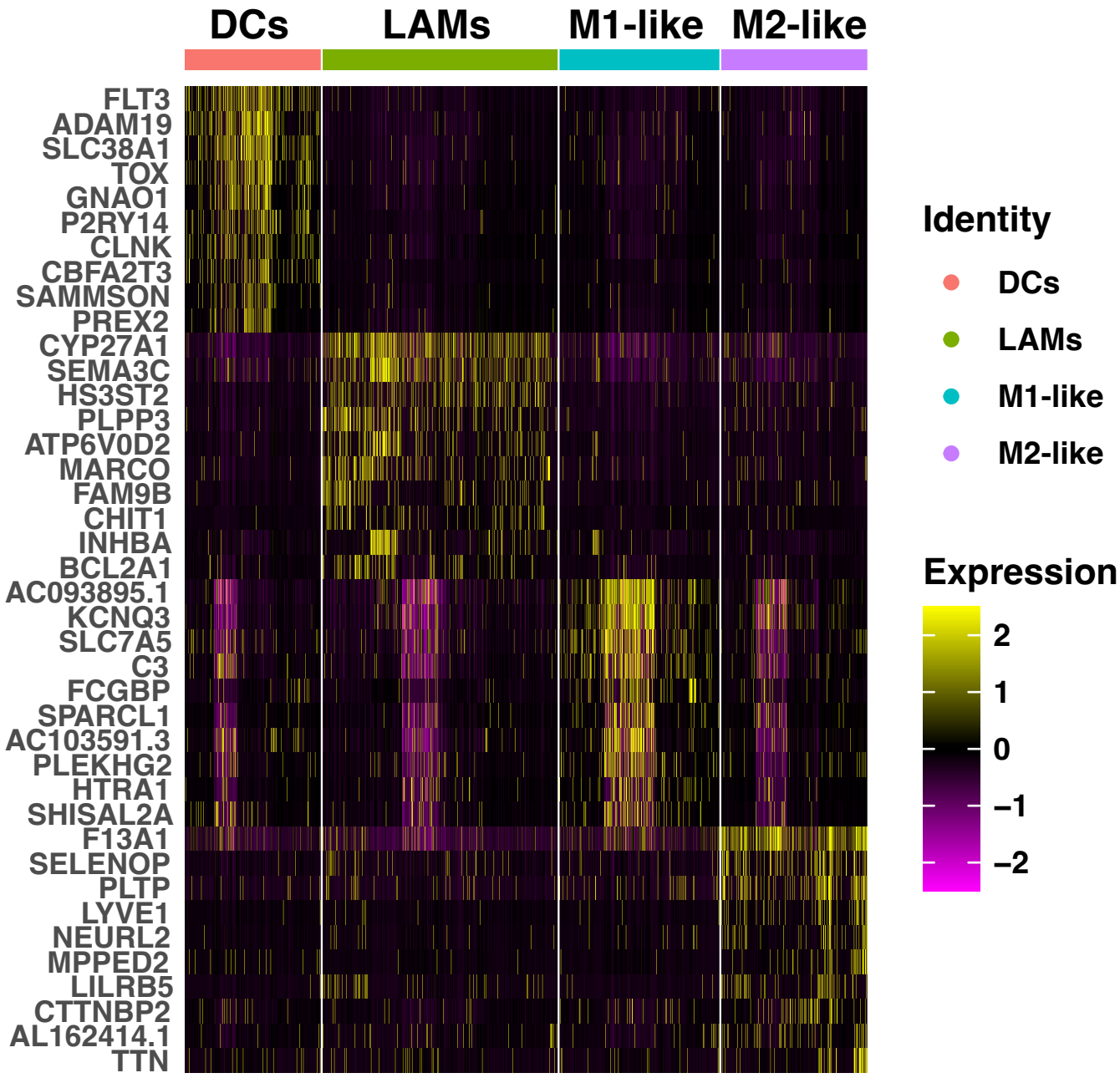
DCs
LAMs
M1-like
M2-like
FLT3
ADAM19
SLC38A1
TOX
GNAO1
P2RY14
CLNK
CBFA2T3
SAMMSON
PREX2
CYP27A1
SEMA3C
HS3ST2
PLPP3
ATP6V0D2
MARCO
FAM9B
CHIT1
INHBA
BCL2A1
AC093895.1
KCNQ3
SLC7A5
C3
FCGBP
SPARCL1
AC103591.3
PLEKHG2
HTRA1
SHISAL2A
F13A1
SELENOP
PLTP
LYVE1
NEURL2
MPPED2
LILRB5
CTTNBP2
AL162414.1
TTN
Identity
DCs
LAMs
M1-like
M2-like
Expression
2
1
0
−1
−2
