# Supplementary material for: Exploratory single‐nucleus multiomics analysis of myeloid cell states associated with neoadjuvant chemotherapy response in pancreatic ductal adenocarcinoma
Source: Clin Transl Med. 2026 Sep 14;16(9):e70810. doi: 10.1002/ctm2.70810 (PMC13574543; doi:10.1002/ctm2.70810)
Supplement: Supplementary file 3 [file CTM2-16-e70810-s004.pdf]

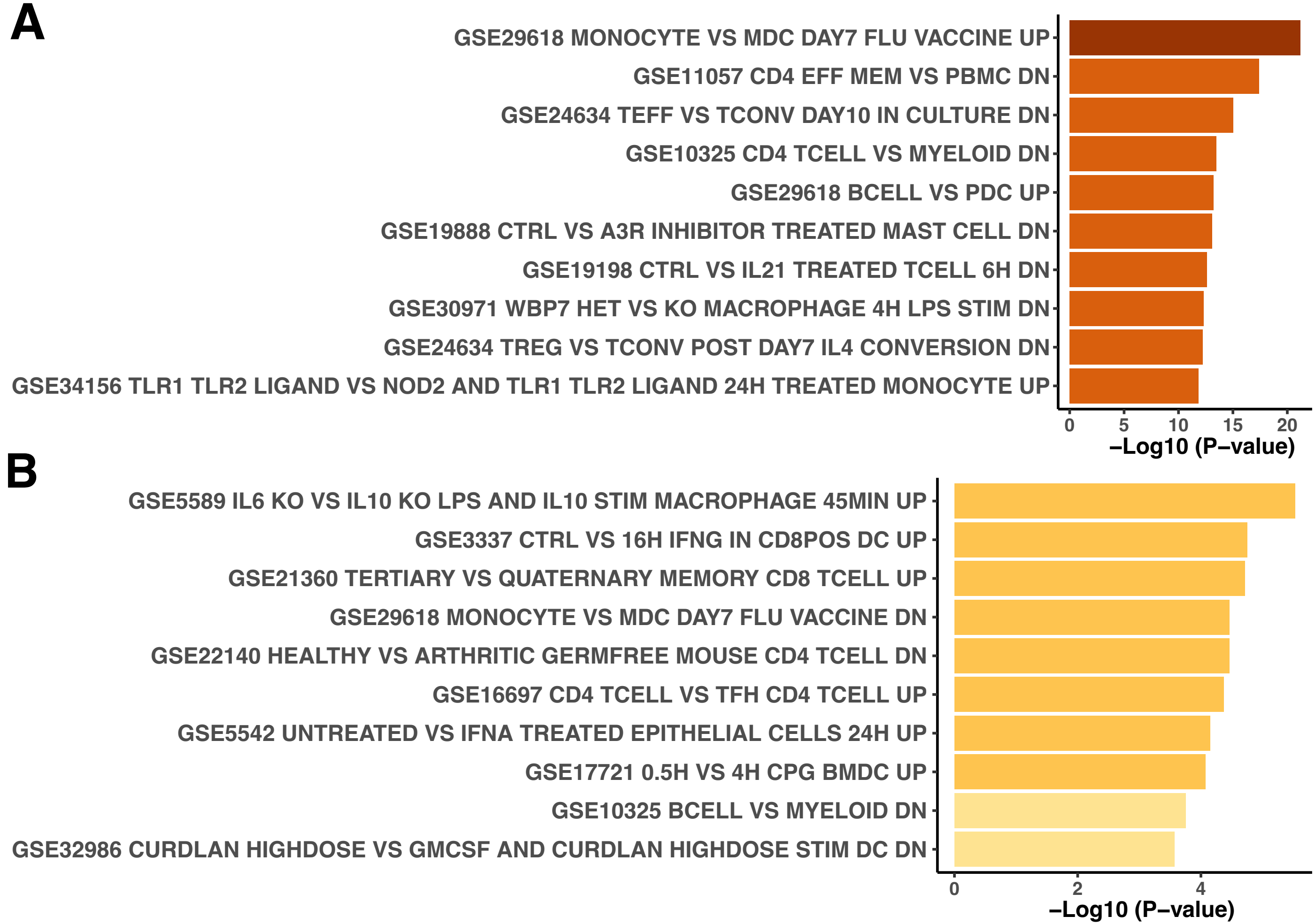
A
GSE29618 MONOCYTE VS MDC DAY7 FLU VACCINE UP
GSE11057 CD4 EFF MEM VS PBMC DN
GSE24634 TEFF VS TCONV DAY10 IN CULTURE DN
GSE10325 CD4 TCELL VS MYELOID DN
GSE29618 BCELL VS PDC UP
GSE19888 CTRL VS A3R INHIBITOR TREATED MAST CELL DN
GSE19198 CTRL VS IL21 TREATED TCELL 6H DN
GSE30971 WBP7 HET VS KO MACROPHAGE 4H LPS STIM DN
GSE24634 TREG VS TCONV POST DAY7 IL4 CONVERSION DN
GSE34156 TLR1 TLR2 LIGAND VS NOD2 AND TLR1 TLR2 LIGAND 24H TREATED MONOCYTE UP
0
5
10
15
20
−Log10 (P−value)
B
GSE5589 IL6 KO VS IL10 KO LPS AND IL10 STIM MACROPHAGE 45MIN UP
GSE3337 CTRL VS 16H IFNG IN CD8POS DC UP
GSE21360 TERTIARY VS QUATERNARY MEMORY CD8 TCELL UP
GSE29618 MONOCYTE VS MDC DAY7 FLU VACCINE DN
GSE22140 HEALTHY VS ARTHRITIC GERMFREE MOUSE CD4 TCELL DN
GSE16697 CD4 TCELL VS TFH CD4 TCELL UP
GSE5542 UNTREATED VS IFNA TREATED EPITHELIAL CELLS 24H UP
GSE17721 0.5H VS 4H CPG BMDC UP
GSE10325 BCELL VS MYELOID DN
GSE32986 CURDLAN HIGHDOSE VS GMCSF AND CURDLAN HIGHDOSE STIM DC DN
0
2
4
−Log10 (P−value)
