# Supplementary material for: Exploratory single‐nucleus multiomics analysis of myeloid cell states associated with neoadjuvant chemotherapy response in pancreatic ductal adenocarcinoma
Source: Clin Transl Med. 2026 Sep 14;16(9):e70810. doi: 10.1002/ctm2.70810 (PMC13574543; doi:10.1002/ctm2.70810)
Supplement: Supplementary file 4 [file CTM2-16-e70810-s007.pdf]

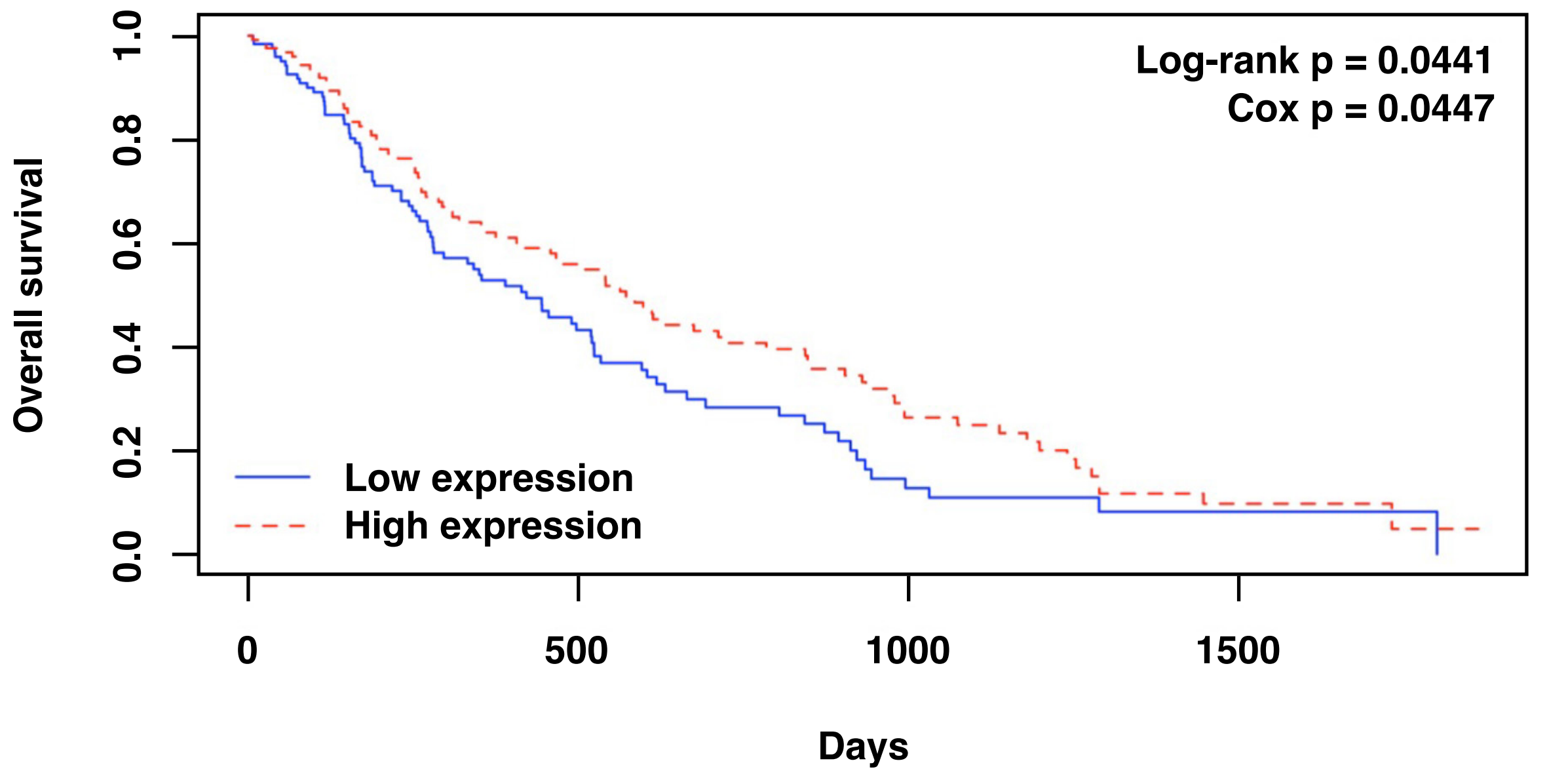

Log-rank p = 0.0441
Cox p = 0.0447
Overall survival
1.0
0.8
0.6
0.4
0.2
0.0
0
500
1000
1500
Days
Low expression
High expression
