# Supplementary material for: Exploratory single‐nucleus multiomics analysis of myeloid cell states associated with neoadjuvant chemotherapy response in pancreatic ductal adenocarcinoma
Source: Clin Transl Med. 2026 Sep 14;16(9):e70810. doi: 10.1002/ctm2.70810 (PMC13574543; doi:10.1002/ctm2.70810)
Supplement: Supplementary file 5 [file CTM2-16-e70810-s008.pdf]

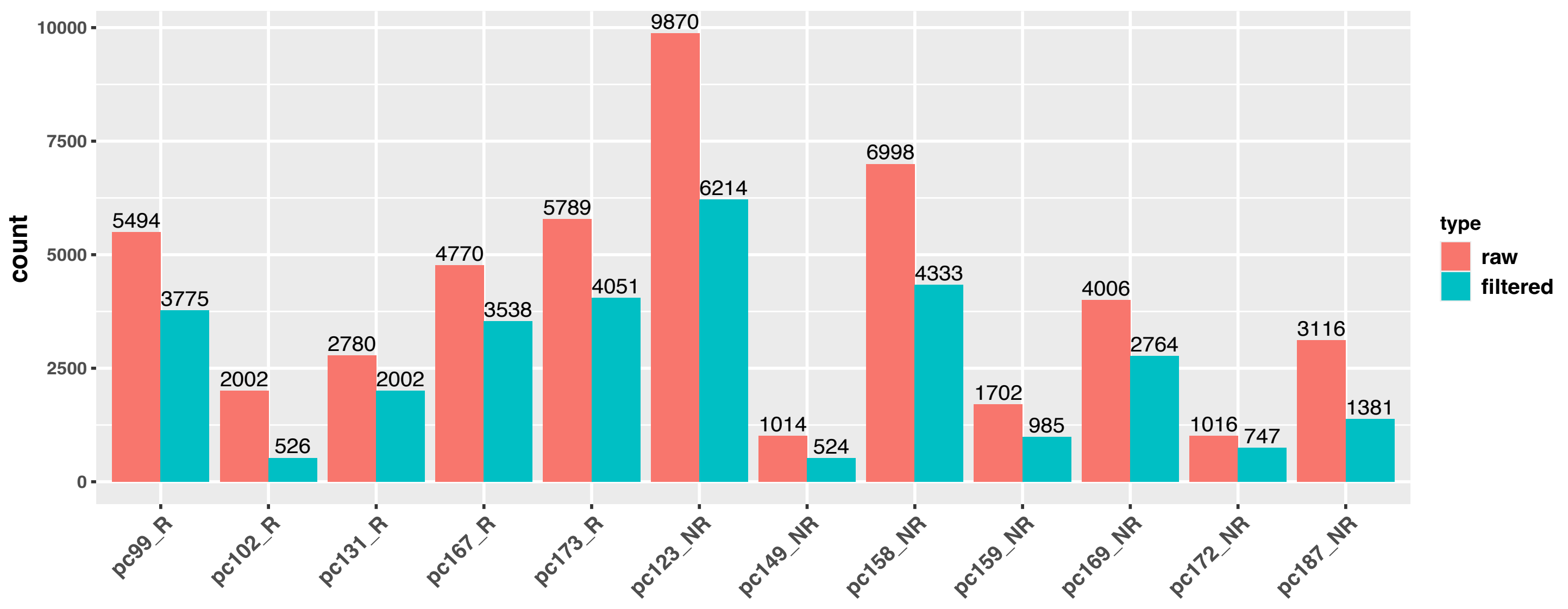
count
10000
7500
5000
2500
0
5494
3775
2002
526
2780
2002
4770
3538
5789
4051
9870
6214
1014
524
6998
4333
1702
985
4006
2764
1016
747
3116
1381
pc99_R
pc102_R
pc131_R
pc167_R
pc173_R
pc123_NR
pc149_NR
pc158_NR
pc159_NR
pc169_NR
pc172_NR
pc187_NR
type
raw
filtered
