# Supplementary material for: Exploratory single‐nucleus multiomics analysis of myeloid cell states associated with neoadjuvant chemotherapy response in pancreatic ductal adenocarcinoma
Source: Clin Transl Med. 2026 Sep 14;16(9):e70810. doi: 10.1002/ctm2.70810 (PMC13574543; doi:10.1002/ctm2.70810)
Supplement: Supplementary file 6 [file CTM2-16-e70810-s003.docx]

**Supplemental Figure Legends**

**Figure S1: Quality Control Filtering displaying the counts of the raw single-nucleus multiome database with a total of 48,557 cells, and a total of 30,840 cells retained for analysis after quality control, as seen in the filtered**

**Figure S2:** **cell type annotation using marker genes in the snMultiome dataset and cell type distribution among the PDAC samples (A) Violin plot showing the expression level of representative marker genes of individual cell types (B) Individual cell type count in the overall dataset retained for downstream analysis (C) Cell type percentage distribution across all samples. (D) Cell type percentage distribution across cohorts, stratified by response type.**

**Figure S3: Enrichment analysis of upregulated and downregulated myeloid genes of the PDAC chemotherapy responders. (A) Top 10 immunologic signatures of the upregulated myeloid genes in the responders. (B) Top 10 immunologic signatures of the downregulated myeloid genes in the responders.**

**Figure S4: Heatmap showing the expression of representative genes of the myeloid subsets.**

**Figure S5: Kaplan–Meier overall survival by 9-gene LAM signature score (PanCAN KYT; median split).** Exploratory overall survival analysis using a scRNA-derived 9-gene LAM signature in the PanCAN Know Your Tumor (KYT) cohort. Kaplan–Meier curves show overall survival (rwOS; days from index sequencing date to death) for primary PDAC tumors from the PanCAN SPARK/KYT molecular profiling program. A 9-gene LAM signature (MARCO, APOC1, AQP9, LPL, ATP6V0D2, ACP5, HS3ST2, CSTB, HTRA4) was derived from the snMultiome dataset using Seurat FindAllMarkers (avg_log2FC > 2, FDR < 0.001, pct.1 > 0.1, pct.2 < 0.05) and selecting the top genes ranked by prevalence gap (pct.1 − pct.2). For each subject, log2(TPM+1) expression values were aggregated into a standardized signature score, and patients (n = 254 primary tumors; 167 deaths) were dichotomized at the cohort median into **High** (red dashed) and **Low** (blue solid) signature groups. The log-rank p-value (0.0441) and univariable Cox proportional hazards p-value (0.0447; HR for High vs Low = 0.73, 95% CI 0.54–0.99) are shown on the plot. Because chemotherapy response annotation and AJCC stage information were limited in the KYT dataset, this analysis is exploratory and should not be interpreted as stage-independent prognostic validations.
